# The Rise of 2D Photothermal Materials beyond Graphene for Clean Water Production

**DOI:** 10.1002/advs.201902236

**Published:** 2020-01-27

**Authors:** Zhongjian Xie, Yanhong Duo, Zhitao Lin, Taojian Fan, Chenyang Xing, Li Yu, Renheng Wang, Meng Qiu, Yupeng Zhang, Yonghua Zhao, Xiaobing Yan, Han Zhang

**Affiliations:** ^1^ Shenzhen Engineering Laboratory of Phosphorene and Optoelectronics SZU‐NUS Collaborative Innovation Center for Optoelectronic Science & Technology International Collaborative Laboratory of 2D Materials for Optoelectronics Science and Technology of Ministry of Education College of Physics and Optoelectronic Engineering Shenzhen University Shenzhen 518060 China; ^2^ Faculty of Information Technology Macau University of Science and Technology Macao 519020 P. R. China; ^3^ Center for Stretchable Electronics and Nanoscale Systems Key Laboratory of Optoelectronic Devices and Systems of Ministry of Education College of Physics and Optoelectronic Engineering Shenzhen University Shenzhen 518060 P. R. China; ^4^ College of Health Science and Environmental Engineering Shenzhen Technology University Shenzhen 518118 China; ^5^ College of Physics and Optoelectronic Engineering Shenzhen University Shenzhen 518060 China; ^6^ State Key Laboratory of Quality Research in Chinese Medicine Institute of Chinese Medical Sciences University of Macau Macao 519020 P. R. China; ^7^ College of Electron and Information Engineering Hebei University Baoding 071002 P. R. China

**Keywords:** 2D photothermal nanomaterials, green technology, photothermal evaporation, solar steam generation, water challenges

## Abstract

Water shortage is one of the most concerning global challenges in the 21st century. Solar‐inspired vaporization employing photothermal nanomaterials is considered to be a feasible and green technology for addressing the water challenge by virtue of abundant and clean solar energy. 2D nanomaterials aroused considerable attention in photothermal evaporation‐induced water production owing to their large absorption surface, strong absorption in broadband solar spectrum, and efficient photothermal conversion. Herein, the recent progress of 2D nanomaterials‐based photothermal evaporation, mainly including emerging Xenes (phosphorene, antimonene, tellurene, and borophene) and binary‐enes (MXenes and transition metal dichalcogenides), is reviewed. Then, the optimization strategies for higher evaporation performance are summarized in terms of modulation of the intrinsic photothermal performance of 2D nanomaterials and design of the complete evaporation system. Finally, the challenges and prospective of various kinds of 2D photothermal nanomaterials are discussed in terms of the photothermal performance, stability, environmental influence, and cost. One important principle is that solutions for water challenges should not introduce new environmental and social problems. This Review aims to highlight the role of 2D photothermal nanomaterials in solving water challenges and provides a viable scheme toward the practical use in photothermal materials selection, design, and evaporation systems building.

## Graphical abstract

The recent progress of two‐dimensional (2D) nanomaterials‐based photothermal evaporation owing to the unique advantages of 2D photothermal nanomaterials, such as the large absorption surface, strong absorption in broadband solar spectrum, and efficient photothermal conversion is reviewed. This Review aims to highlight the role of 2D photothermal nanomaterials in solving the water challenges through employing the abundant and clean solar energy.

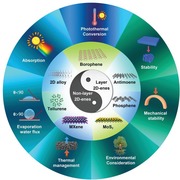

## 1 Introduction

Water is the basic need of our daily life and has become one of the most severe global challenges in the 21st century. Much effort has been devoted to this field in order to deliver better solutions.^1^, ^2^, ^3^ Light‐to‐heat, namely, photothermal conversion, an original ancient strategy in utilizing solar energy involving converting solar radiation into heat by using photothermal materials for beneficial usage.^4^, ^5^, ^6^, ^7^ Due to its easy operability and especially high light conversion efficiency, it has obtained notable research interest in recent years and find itself suitable for diverse applications, including seawater desalination,^8^, ^9^, ^10^ steam generation,^11^, ^12^, ^13^, ^14^ energy production,^15^, ^16^, ^17^ and cancer therapy,^18^, ^19^, ^20^ which hold the great potential in solving the current urgent global challenges in energy, health, and environment. The photothermal effect based on nanomaterials arouses a particular interest owing to the precise heat location to an appointed region at the nanoscale.^7^, ^21^, ^22^, ^23^, ^24^ Additionally, the adjustable surface and structure morphology contribute to quantum confinement effects and localized surface plasmonic resonance (LSPR), which further enhances the photothermal performance.^25^, ^26^, ^27^

Since the discovery of graphene,^28^, ^29^ 2D nanostructured materials, as a rising family member of nanomaterials, have received extensive attention owing to their unique morphological and physicochemical properties.^30^, ^31^, ^32^, ^33^, ^34^, ^35^, ^36^ First, the thickness‐dependent bandgap of 2D materials contributes to wide absorption band corresponding to solar spectrum. Visible and infrared light takes up 97% of the solar spectrum. The absorption of 2D materials happens to correspond to these spectra owing to their unique thickness‐dependent bandgap. For example, the bandgap of black phosphorus (BP) can be tuned from 0.3 to 2 eV through fabricating the BP nanosheets with different layers, which just cover the solar spectrum band from visible light (≈600 nm) to far infrared light (4000 nm). Moreover, kinds of 2D materials possess different bandgaps. Typical transition metal dichalcogenides (TMDs) materials hold a larger bandgap (≈1–2.5 eV) and can be used for absorbing short‐wave solar light. Combination of different 2D materials can realize the full soar spectral absorption. Second, efficient photothermal conversion can be realized by 2D materials. MXene is a typical kind of 2D material. By using a delicately designed light heating system, a perfect energy conversion was found with photothermal conversion efficiency (PTCE) to be up to 100%,^37^ showing the promising potential of 2D MXene for photothermal evaporation applications. Third, 2D materials can be efficiently utilized for low‐cost photothermal device. Owing to the ultrathin thickness of 2D materials (less than 1 nm), a small quantity can be extended to a large area and used for fabricating large‐area solar absorption materials. For example, graphene has large specific surface area of 2630 m^2^ g^−1^, which can tremendously decrease the cost of solar evaporation device and promote its practical application. Moreover, the large surface area of 2D nanosheets provides an expansive platform for tailoring the physicochemical properties and functionalities.^38^ Fourth, the 2D nanomaterials can be directly obtained from their bulk counterpart through top‐down fabrication no matter how the bulk materials are layered or not,^39^, ^40^, ^41^ differing from the fabrication of other low‐dimensional nanomaterials, whose synthesis mainly depends on the bottom‐up growth.^42^, ^43^ Therefore, the bulk crystal phase can be completely inherited. Fifth, the work on photothermal evaporation has been widely reviewed. Gao et al. comprehensively reviewed the photothermal materials for evaporation application, including metallic materials, semiconductors, and carbon‐based materials.^44^ Zhu et al. surveyed the different solar energy conversion materials for wide applications in water purification, seawater desalination, and energy generation.^45^ Different from these two Reviews, Chen et al. emphasized the challenges and further developments for solar evaporation.^46^ Tao et al. highlight the advantage of interfacial evaporation than conventional bulk heating‐based evaporation in their Review.^47^ Similarly, Zhou et al. focus on the interfacial mechanism and structure design.^48^ However, none of them focuses on the 2D materials‐based photothermal evaporation.

The 2D nanosheets can be further employed for preparing novel functional blocks,^49^ porous membrane,^37^ etc. 2D photothermal materials have experienced their wide investigations in photothermal cancer therapy.^50^, ^51^, ^52^, ^53^ For photothermal evaporation, extensive research has been reported on graphene‐based materials,^13^, ^54^, ^55^, ^56^, ^57^, ^58^, ^59^, ^60^, ^61^, ^62^, ^63^, ^64^, ^65^, ^66^ and has been reviewed.^67^, ^68^, ^69^ Recently, the development of other 2D nanomaterials inspired by graphene brings the fresh blood to 2D nanomaterials‐based photothermal evaporation recently and has not been summarized systematically.

**Figure 1:**
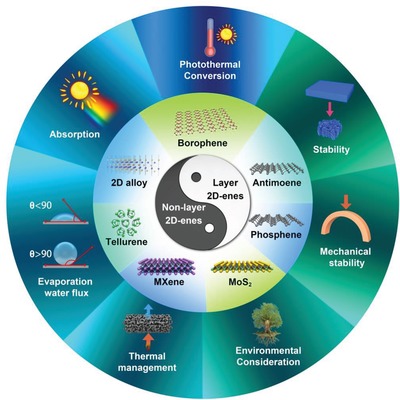
Schematic illustration of the 2D photothermal nanomaterials‐based photothermal evaporation.

Herein, we review the recent development in 2D nanomaterials‐based photothermal evaporation beyond graphene‐based nanomaterials (**Figure** **1**). We firstly illustrate the mechanism of photothermal conversion for water evaporation. Then, the development of 2D photothermal nanomaterials is reviewed. Furthermore, both the strategies from materials to system are presented. For materials design, various strategies in enhancing the light absorption and photothermal efficiency are discussed, and the methods for optimizing the whole photothermal evaporation system are further summarized and compared. Finally, the challenges, perspectives, and conclusions for the practical solar‐driven evaporation are discussed. Given the beginning research of 2D nanomaterials based photothermal evaporation, the purpose of this Review is to point out the advantage by employing these unique 2D materials in solar light conversion and provide a guide for further designing evaporation systems.

## 2 Fundamental Mechanisms of Photothermal Conversion

Considering the different light–matter interaction mechanisms in electromagnetic radiation, two kinds of 2D nanomaterials can be divided, including the metallic materials with localized plasmonic heating and semiconductors with nonradiative relaxation. Both mechanisms can contribute efficient photothermal conversion.

### 2.1 Plasmonic Localized Heating in Metallic 2D Photothermal Agents

**Figure 2:**
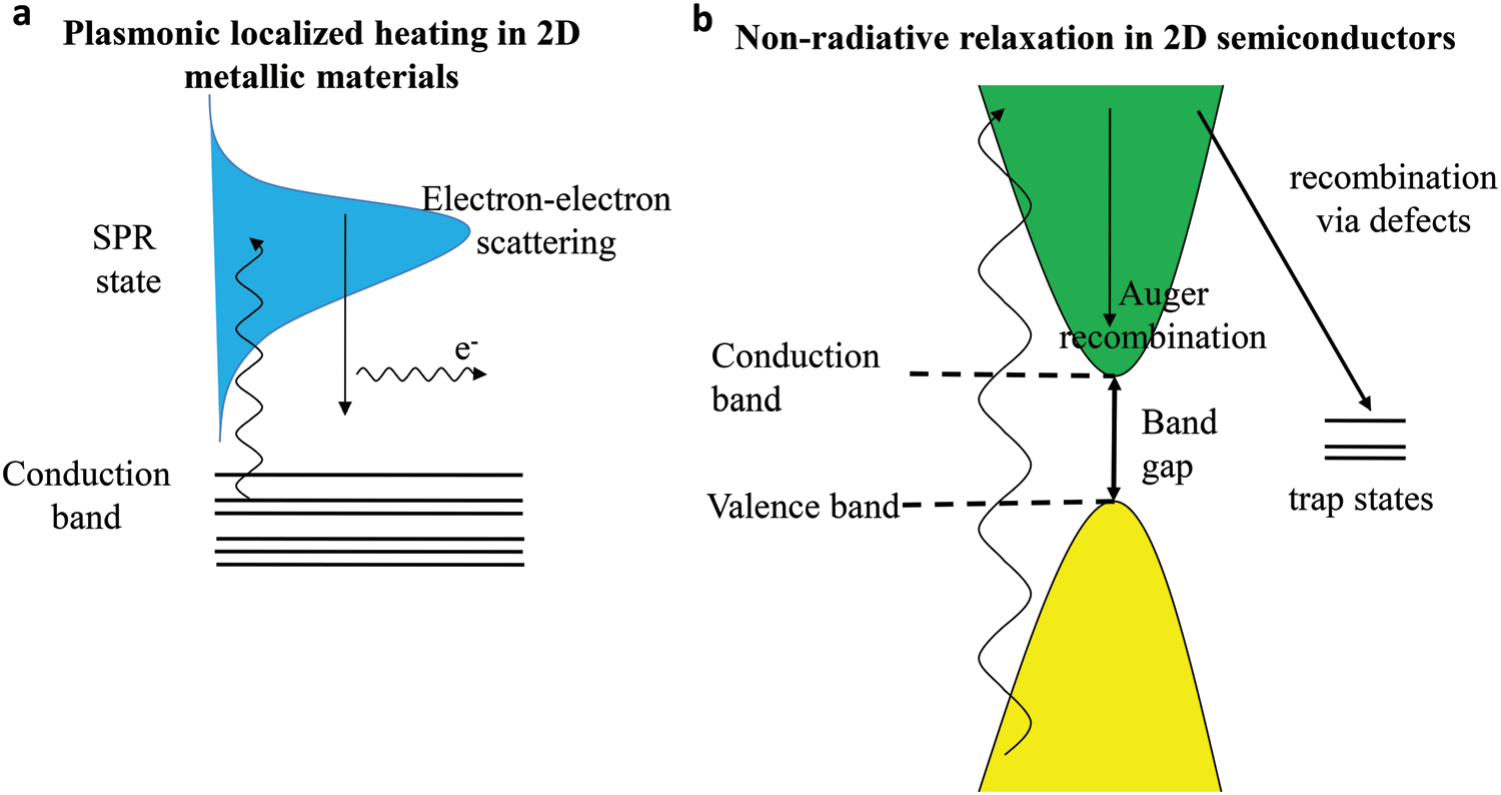
a,b) Different photothermal mechanisms for 2D metallic materials and 2D semiconductors.

The investigation about plasmonic photothermal effect starts since 2002,^70^ and mainly focuses on biomedical application, for instance, the photothermal cancer therapy or smart drug delivery.^71^, ^72^ The noble metals nanomaterials, such as gold nanoparticles, are classic plasmonic photothermal materials, which are observed to show brilliant colors owing to the intrinsic LSPR of metals. The LSPR presented by newly emerging 2D plasmonic materials, such as MXenes, endows their strong absorption.^73^ When the incident photon frequency provide a good match with the inherent frequency of electrons on the metal surface, the LSPR occurs with the photon‐induced coherent oscillation of electrons,^74^ resulting in near‐field enhancement, thermal electron generation, and final photothermal conversion (**Figure** **2**a).^75^ To be more specific, the light irradiation induces the oscillation of electron from the occupied state to unoccupied state and creates the hot electron, leading to a thermal charge‐carrier distribution. After thermalization, hot electron cools down through transferring energy to the lattice phonon, which results in a temperature increase.^70^ The main factors influencing the shape and position of the LSPR band are particle shape and size,^76^ coulombic charge,^77^ and dielectric constant in nanoparticles.

### 2.2 Nonradiative Relaxation in Semiconducting 2D Photothermal Agents

For semiconducting materials, a strong absorption occurs at the wavelength matching the bandgap energy. When irradiated by light, electron–hole pair is formed with similar energy corresponding to bandgap. The bandgap of 2D photothermal nanomaterials differs according to the different species. For one 2D photothermal nanomaterial, the bandgap varies with the size of nanosheets, such as the tunable bandgap range of phosphorene from 0.3 to 2 eV,^78^ which can be employed to capture the solar light with different wavelengths. Then, the stimulated electrons fall back to the low energy states and the energy is released through radiating photons, or nonradiative phonons in the interaction with defects or surface dangling bonds.^79^ In nonradiative mode, the heat is produced when the phonon interacts with the lattice, establishing a temperature gradient based on the optical absorption and electron–hole recombination feature. Consequently, the mechanism of photothermal effect in semiconductors is the optical‐stimulated electron diffusing and recombining carriers (Figure 2b).

## 3 Current Developments of 2D Photothermal Nanomaterials for Efficient Water Vaporization

Numerous investigations have been reported on enhancing the photothermal performance though seeking for new 2D photothermal nanomaterials or modification of already‐known 2D nanomaterials. The main 2D nanomaterials beyond graphene include MXenes, tellurene, TMDs, transition metal oxides (TMOs), and 2D layered alloy. The large family of MXenes and TMDs may provide more potential for photothermal water vaporization but only a few members have been investigated up to now.

### 3.1 MXene

MXene, as early transition metal carbides and nitrides, is firstly introduced into the 2D family by Gogotsi's group in 2011.^80^ It is prepared through selectively etching the A layer from M*_n_*_+1_AX*_n_* phases, where M represents an early transition metal, such as Ti, Ta, Nb, Mo, Zr, Cr, and so on, X represents C and/or N, and A is a group IIIA or IVA element.^81^, ^82^ The thickness of an MXene nanosheet is normally less than 1 nm, while its lateral dimension can be up to micrometers with a large aspect ratio. Thanks to the unique fabrication method, MXenes hold abundant functional groups on the surface, such as fluorine, hydroxyl, or oxygen. Thus, MXenes uniquely possess both the excellent conductivity of early transition metal and the hydrophilic property of their fluorine/oxygen/hydroxyl‐terminated surfaces, which enables MXenes many exciting applications,^83^ including supercapacitors,^84^, ^85^ battery,^86^, ^87^ catalysis,^88^, ^89^ molecular separation,^90^ biomedical application,^2^, ^91^, ^92^ seawater desalination,^83^ etc.

**Figure 3:**
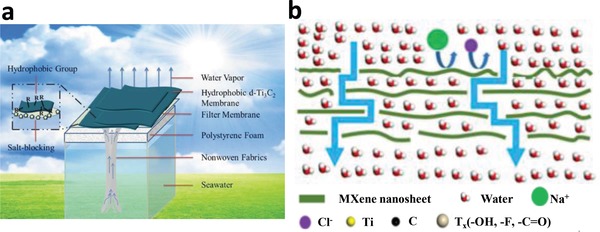
a) Schematic illustration of the photothermal evaporation based on the hydrophobic d‐Ti_3_C_2_ membrane. Reproduced with permission.^93^ Copyright 2018, Royal Society of Chemistry. b) Pervaporation desalination using hydrophilic MXene. Reproduced with permission.^94^ Copyright 2017, Elsevier.

Since the discovery of Ti_3_C_2_ MXene,^80^ numerous kinds of MXenes were successively investigated, such as the Nb_2_C, Ta_4_C_3_, Mo_2_C, etc. Excellent photothermal performance has been found in Ti_3_C_2_, Nb_2_C, and Ta_4_C_3_ and they have been employed for photothermal cancer therapy in the biomedical field,^37^, ^91^, ^92^ and also for photothermal evaporation. The Ti_3_C_2_ MXene was shown to possess both a high internal PTCE of 100% and photothermal evaporation efficiency (84%) through reasonable choosing heat barrier.^37^ Through comparison of hydrophobic and hydrophilic Ti_3_C_2_ membrane, it is found that the hydrophobic membrane can avoid the salt‐blocking problem and keep a long and stable evaporation (**Figure** **3**a).^93^ Conversely, the pervaporation membrane prefers the hydrophilic property to keep salt rejection and high water flux (Figure 3b).^94^ The unique advantage of MXenes is their rich surface groups, which is beneficial for the wetting surface modification.

### 3.2 Tellurene

Te, as a typical chalcogen element, possesses some distinct properties, such as semiconducting,^95^ topological,^96^ and thermoelectric,^97^ which makes it promising for applications in optoelectronics,^98^, ^99^ electronics devices,^100^, ^101^, ^102^ and biomedical field.^103^, ^104^, ^105^, ^106^ Similar to MXene, the photothermal effect of Te nanomaterials has been investigated for photothermal cancer therapy.^107^, ^108^ Owing to the ultranarrow bandgap of 0.35 eV of Te semiconductor, both a strong NIR absorbance and high PTCE are obtained for Te nanorods.^107^ The Te nanodots encapsulated by hollow albumin nanocages can also serve as the photothermal agent with high resistance to photobleaching.^108^

The research on Te nanomaterials based photothermal evaporation is just beginning. Ma et al. fabricated a broadband absorber by using different sized Te nanoparticles, which can absorb more than 85% solar light spanning the entire solar spectrum.^109^ Besides, the solar conversion performance has surpassed that of layered BiInSe foam,^110^ Ge nanoparticles,^111^ and Al nanoparticles.^112^ Temperature of the absorber gives a dramatic increase from 29 to 85 °C within 100 s upon irradiation of sunlight. The evaporation performance of the dispersion of Te nanomaterials in water can be enhanced by three times that of pure water under the same simulated solar radiation.

### 3.3 TMDs and TMOs

TMDs, expressed as MX_2_, in which M represents transition metal element of Mo, W, Sn, Hf, Zr, etc., and X represents chalcogen atom of S, Se, and Te, reveal an excellent performance in electrical, optical, chemical, and mechanical properties.^113^, ^114^ Through the van der Waals forces, a layer of transition metal atoms is sandwiched by two layers of chalcogen atoms. As a semiconductor, TMDs possess the advantages of sizable bandgap, relatively high carrier mobility, stability in air, and so on.^115^, ^116^

MoS_2_, as a typical TMD, has aroused great attention due to the significantly unique mechanical,^117^ electrical,^118^, ^119^, ^120^, ^121^ and biological properties.^122^, ^123^, ^124^, ^125^ Correspondingly, numerous applications have been demonstrated in catalysis,^126^, ^127^ electronics,^128^, ^129^, ^130^ energy,^131^, ^132^ and biomedical‐related fields.^133^, ^134^, ^135^, ^136^ The environmental applications of 2D MoS_2_ are also expected. Actually, the bulk MoS_2_, which exists as mineral molybdenite in nature, has been applied as environmental adsorbents^137^ and catalysts^138^ in a long term. However, the environmental applications of MoS_2_ are always limited by the synthesis of monolayer or few‐layer MoS_2_ nanosheets, which hold unique 2D nanosized properties. Recently, the development of advanced technologies in obtaining the monolayer and large‐scale MoS_2_ nanosheets provides a new potential.^139^, ^140^ Following the original work, the synthesis methods,^127^, ^141^, ^142^ physical and chemical tuning,^143^ and functionalization^144^ have been reported, and novel environmental applications based on photothermal effect of MoS_2_ are revealed.^145^, ^146^

**Figure 4:**
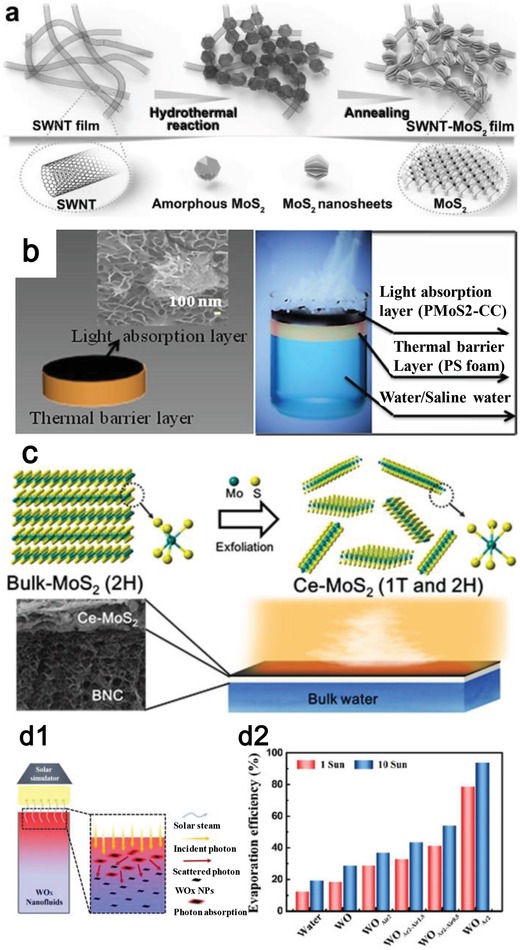
a) Synthesis of SWCNT‐MoS_2_ system. Reproduced with permission.^151^ Copyright 2017, WILEY‐VCH. b) Schematic illustration of MoS_2_‐cotton cloth system. Reproduced with permission.^156^ Copyright 2018, American Chemical Society. c) Chemically exfoliated (ce)‐MoS_2_ for photothermal evaporation. Reproduced with permission.^157^ Copyright 2018, Elsevier. d1) Schematic of the WO*_x_* nanofluids‐based device for photothermal evaporation and d2) the high evaporation efficiency. Reproduced with permission.^159^ Copyright 2018, Elsevier.

Photothermal effect of MoS_2_ has been reported for bacteria‐infected wound therapy,^147^ infrared light harvesting,^148^ and photothermal cancer therapy.^149^, ^150^ For photothermal evaporation, Yang et al. designed an ultrathin and porous composite film using MoS_2_ and single‐walled carbon nanotubes (SWCNTs) (**Figure** **4**a)_._ 151 SWCNTs, as a typical 1D nanomaterial, possess both excellent thermal transport properties^152^, ^153^ and high mechanical strength.^152^, ^154^ Moreover, the hydrophobic and ultrathin nature of SWCNTs endows them with super permeability of water vapor. Therefore, the SWCNTs can be used to combine with 2D nanomaterials, such as the typical MoS_2_, in order to obtain a high‐performance evaporation system with enhanced photothermal properties, superior mechanical strength, high stable performance, and high‐producing vapor.^151^, ^155^

Rather than using SWCNTs, Guo et al. fabricated a new hybrid of PEGylated MoS_2_ with cotton cloth for preparing clean water (Figure 4b).^156^ The cotton cloth is chosen owing to its numerous advantages, such as large area, unobstructed water transport, high‐temperature resistance, and also the low cost. The attachment of MoS_2_ and cotton cloth is achieved through the self‐growth technique in a hydrothermal process, and the hydrophilic surface is further optimized through polyethylene glycol (PEG) modification. Both a high absorption covering the solar spectrum and high stability can be achieved.

The solar absorber of MoS_2_ in both the two reports is obtained through the bottom‐up method. In Chou's study, MoS_2_ was proved to be fabricated by a top‐down method, i.e., chemically exfoliation.^116^ Then, Ghim et al. used the chemically exfoliated MoS_2_ to conduct the photothermal evaporation (Figure 4c).^157^ It is interesting to find that there is a transition from 2H phase (trigonal prismatic coordination) to 1T phase (octahedral coordination) in the chemical exfoliation process and this transition can enhance the light absorption. Moreover, the biocompatibility of chemically exfoliated MoS_2_ is evaluated, and the toxicity is lower than graphene oxide (GO). The combined material contributes to solar evaporation efficiency of 81% under light energy of 5.35 kW m^−2^. These solar evaporation systems prove MoS_2_ can be an excellent solar absorber for converting solar energy into heat.

TMOs, cousins of TMDs, also show its promise for photothermal conversion. Typical TMOs include nonstoichiometric MoO*_x_*,^158^ and mesoporous black TiO_2_, which have been investigated regarding their high photothermal conversion ability owing to the strong light absorption and LSPR. Ming et al. demonstrated an efficient photoabsorber based on tungsten oxide (WO*_x_*) nanosheets for the solar steam generation owing to its tunable LSPR effect (Figure 4d).^159^ Through the introduction of surface oxygen vacancies, the morphologies of the WO*_x_* nanosheet can be elaborately designed and the evaporation efficiency is up to ≈78.6% upon sun irradiation. This study provides a guide for designing the plasmonic 2D nanomaterials in enhancing solar absorption.

### 3.4 2D Layered Alloy Material

**Figure 5:**
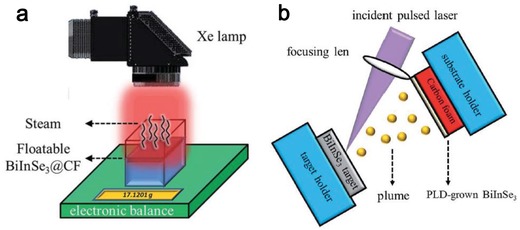
a) Schematic of the photothermal evaporation system based on BiInSe_3_@CF, and b) the experimental setup of the PLD fabrication of BiInSe_3_. Reproduced with permission.^162^ Copyright 2018, Royal Society of Chemistry.

2D layered alloy materials (2DLAMs) have also been considered for photothermal evaporation owing to their abundant intermediate band (IB) states,^160^ tunable optical properties, strong light–matter interaction, and superior stability. BiInSe_3_ is chosen as photothermal agent owing to the abundant IB states from interleaved electron structures of In_2_Se_3_ and Bi_2_Se_3_.^161^ 2D BiInSe_3_ alloy based evaporation systems have been built, one is the BiInSe_3_‐coated nickel foam (BiInSe_3_@NF) and the other is BiInSe_3_‐coated carbon foam (BiInSe_3_@CF) (**Figure** **5**a).^110^, ^162^ In the fabrication of BiInSe_3_, pulsed laser deposition (PLD) technique has been employed as a clean, simple, and efficient method without introducing precursors and catalysts (Figure 5b). Moreover, the PLD technique is compatible to the substrate of NF and CF, which exhibits its absolute advantage compared with chemical vapor deposition (CVD). Thanks to the efficient phonon emission owing to the existence of IB states and the scattering‐based light trapping in rough substrate, a high photothermal evaporation performance is obtained with evaporation rate of 0.83 kg m^−2^ h^−1^ upon the irradiation power density of 1 sun. Consequently, the well‐designed floatable evaporator based on 2D alloying materials shows its great potential in achieving high‐performing photothermal evaporation.

## 4 Design Strategies of 2D Photothermal Nanomaterials for High Photothermal Performance

As discussed above, two main properties evaluating photothermal performance are absorption and photothermal conversion. Tremendous efforts have been devoted on enhancing absorption but less work on photothermal conversion. The specific strategies will be described in this section.

### 4.1 Criteria for Efficient Photothermal Conversion

The seeking of high‐performing photoabsorbers with strong absorption and high photothermal conversion efficiency is of particular importance for realizing efficient light utilization.

#### 4.1.1 Light Absorption

It is expected that the absorber can absorb most of the light energy. To evaluate the absorption ability, the extinction coefficient (*k*) is characterized through Lambert–Beer law

(1) k=A(λ)/LC

where *A* is the wavelength (λ)‐dependent absorbance, *L* is the traversing length of light (in cm), and *C* is the concentration of 2D nanomaterials (in g L^−1^).

#### 4.1.2 Light–Heat Conversion

The basic principle is that the absorbed light should be converted to heat to the maximum, rather than other forms of energy. To evaluate this ability, the PTCE (η) is defined. In Roper's report,^163^ the energy equilibrium for a complete photothermal system in solution is

(2) $$mC_{\text{p}}\frac{\text{d}T}{\text{dt}}\;=\;Q_{\text{I}}+Q_{\text{s}}-Q_{\text{loss}}$$

where *m*, *C*_p_, and *T* are the mass, heat capacity, and temperature of the photothermal system, respectively, *Q*_I_ is the energy input of the whole system, *Q*_s_ is the energy dissipated by the container itself, and *Q*_loss_ is external heat flux.

The light‐induced heat source term, *Q*_I_ represents heat produced by electron–phonon relaxation on the surface of the 2D photothermal nanomaterials under the light irradiation

(3) $$Q_{\text{I}}=I(1-10^{-A_{\lambda }})\eta$$

where *I* is incident light energy, *A_λ_* is the absorbance at a specific wavelength, and η is the PTCE.

*Q*_loss_ is a linear function to the temperature of photothermal system

(4) $$Q_{\text{loss}}\;=\;hA(T-T_{\text{surr}})$$

where *h* is heat transfer coefficient, *A* is heat transfer area of the photothermal system, and *T* and *T*_surr_ are the temperatures of the photothermal system and the surrounding temperature.

Since the heat loss (*Q*_loss_) increases with increased temperature (Equation (4)), the photothermal system would reach a balance

(5) $$Q_{\text{I}}+Q_{\text{s}}\;=\;Q_{\text{loss‐equ}}=\;hA(T_{\text{equ}}-T_{\text{surr}})$$

where *Q*_loss‐equ_ is the conductive heat escaping from photothermal system when it reaches to a balanced state. *T*_equ_ is the equilibrium temperature. By substituting Equation (3) into Equation (5), the PTCE (η) can be calculated

(6) $$\eta =\frac{hA(T_{\text{equ}}-T_{\text{surr}})-Q_{\text{s}}}{I(1-10^{A_{\lambda }})}$$

In order to obtain the parameter of *hA*, a heat driving force, θ, is introduced through

(7) $$\theta =\frac{T-T_{\text{surr}}}{T_{\text{equ}}-T_{\text{surr}}}$$

and a time constant for heat transfer is

(8) $$\tau_{\text{s}}=\frac{mc}{hA}$$

Substituting Equations (7) and (8) into Equation (3), we obtain

(9) $$\frac{\text{d}\theta }{\text{d}t}\;=\;\frac{1}{\tau_{\text{s}}}\;\left(\frac{Q_{\text{I}}+Q_{\text{s}}}{hA(T_{\text{equ}}-T_{\text{surr}})}-\theta \right)$$

When the light irradiation is off, the photothermal system cools down and *Q*_I_ + *Q*_dis_ = 0. Then Equation (9) can be simplified to

(10) $$\text{d}t=-\tau_{\text{s}}\frac{\text{d}\theta }{\theta }$$

After integrating, Equation (10) turns to

(11) $$t=-\tau_{\text{s}}\;\text{ln}\theta$$

#### 4.1.3 External Photothermal Conversion Efficiency

The normally evaluated index of PTCE is actually the internal photothermal efficiency, indicating how much the absorbing light can be converted into heat. Then the true photothermal conversion ratio of input light energy to output thermal energy, reflecting the actual photothermal ability, has not been characterized. Given this issue, a new parameter, namely, external photothermal conversion efficiency (ePTCE, γ), is defined to comprehensively evaluate the photothermal performance in both considering the absorption ability and photothermal conversion, which is

(12) γ=k⋅η

For monoelemental class of 2D nanomaterials, termed 2D‐Xenes, strong absorption for phosphorene induces the low photothermal conversion while the weak absorption in borophene, antimonene, and tellurene leads to higher PTCE. However, phosphorene obtains the best photothermal performance by the overall consideration through ePTCE (**Table** **1**). As atomic number increases or decreases in comparing with phosphorene, the ePTCE decreases.

**Table 1:** The comparison of photothermal performance for Xenes

Xenes	B^313^	P^320^	Sb^18^	Te (This work)
Group	IIIA	VA	VA	VIA
Period	2	3	5	5
Atomic number	5	15	51	52
Extinction coefficient [L g^−1^ cm^−1^]	∼2.5	14.8	5.6	4.9
PTCE	42.5%	28.4%	45.5%	44.7%
ePTCE [L g^−1^ cm^−1^]	1.06	4.2	2.5	2.2

In MXene family, the photothermal performance is dependent on the component of early transition metal (**Table** **2**). As the atomic number of early transition metal increases, the extinction coefficient, representing the absorption ability, firstly increases and then decreases, while the PTCE always increases and the ePTCE, reflecting the true photothermal conversion ability, firstly rises and then drops, indicating the best photothermal performance occurring in Nb_2_C MXene, which composes of an early transition metal with moderate atomic number.

**Table 2:** The comparison of photothermal performance for MXenes

MXene	Ti_3_C_2_ 2	Nb_2_C^92^	Ta_4_C_3_ 321
Atomic number of metal	22	41	73
Group of metal	IVB	VB	VB
Period of metal	4	5	6
Extinction coefficient [L g^−1^ cm^−1^]	25.2	36.4	4.06
PTCE	30.6%	37.6%	44.7%
ePTCE [L g^−1^ cm^−1^]	7.7	13.7	1.8

For transition metal dichalcogenides (TMDs), the photothermal performance differs depending on the component of early transition metal and chalcogen elements (**Table** **3**). As both the atomic number of metal or chalcogen element increases (from MoS_2_ to WS_2_, or from MoS_2_ to MoSe_2_), the extinction coefficient would decrease, and the PTCE would increase. The overall photothermal performance evaluated by ePTCE obtains a similar level.

**Table 3:** The comparison of photothermal performance for TMDs

TMDs	MoS_2_	WS_2_	MoSe_2_ 322	WSe_2_ 323
Atomic number	42(Mo)/16(S)	74/16	42/34	74/34
Extinction coefficient [L g^−1^ cm^−1^]	28.4^122^	23.8^324^	17.4	–
PTCE	27.6%^18^	32.8%^325^	46.5%	35.1%
ePTCE [L g^−1^ cm^−1^]	7.8	7.8	8.1	–

### 4.2 Design of 2D Photothermal Nanomaterials for Strong Absorption

A strong absorption is the first and foremost key parameter to guarantee a high photothermal performance and thus many strategies have been employed to improve the absorbance through defect modulation,^159^ combination of plasmonic and all‐dielectric properties,^109^ introduction of IB states in alloying materials,^110^, ^162^ phase transition,^156^ and hybrid design.^151^, ^156^

#### 4.2.1 Defect Modulation

**Figure 6:**
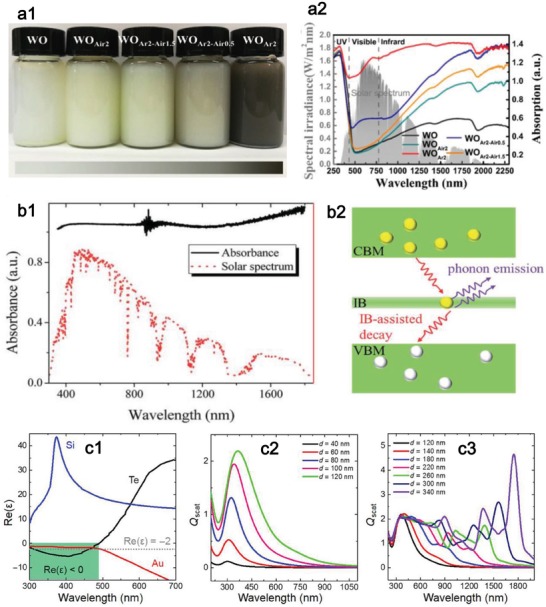
a) Introduction of oxygen vacancies for enhancing absorption. Reproduced with permission.^159^ Copyright 2018, Elsevier. a1) Digital picture of WO*_x_* solution with different defective level. a2) Standard solar spectrum and corresponding absorption spectrum of the defective WO*_x_*. b) IB‐assisted absorbance in BiInSe_3_. Reproduced with permission.^110^ Copyright 2017, Royal Society of Chemistry. b1) Absorption spectrum of the BiInSe_3_ device and solar spectrum. b2) Illustration of the IB‐assisted decay for photoinduced electrons though electron–phonon interactions. c) Dual‐resonance mode for Te nanomaterials. Reproduced with permission.^109^ Copyright 2018, American Association for the Advancement of Science. c1) Comparison of the real part of permittivity of Te nanomaterials with Si (an all‐dielectric material) and Au (plasmonic material). c2) Plasmonic‐like scattering of Te nanomaterials with size less than 120 nm. c3) All‐dielectric scattering of Te nanomaterials with size larger than 120 nm.

TMOs are well known as their excellent chemical stability and large bandgap. For instance, TiO_2_ and ZnO have a wide optical bandgap of 3.2–3.4 eV, which make them only absorb 4% of light in solar spectrum. Fortunately, WO_3_, MoO_3_, and V_2_O_5_ possess tunable optical absorption from NIR to visible regions through tailoring the particle size.^164^, ^165^, ^166^ Besides, good electron transport properties can assuage the fast charge recombination in TMOs.^167^ Therefore, appropriate design of TMOs‐based compounds is promising for solar vapor generation. The nanostructured design plays an important part in light absorption ability. Through introducing oxygen vacancies, the defective WO*_x_* were obtained.^159^ The outcome of introducing oxygen vacancies is the clear enhanced absorption, as proved by the digital picture (**Figure** **6**a1), which shows the distinct color variation from white to black as the oxygen vacancies increase from WO (WO_3_) to WO_Ar2_ (Figure 6a2). The introduced oxygen vacancies contribute to efficient LSPR and thus the 2D WO*_x_* show intense and broadband light absorption covering the whole solar spectrum. This study shows that the light‐harvesting ability of 2D plasmonic materials can be enhanced through introducing defect effect.

#### 4.2.2 Bandgap Adjustment through IB States

The relatively large bandgap of some 2D materials reduces their light absorption in the long‐wavelength solar spectrum. Alloying materials can be used to adjust the bandgap and bring IB states,^160^, ^168^, ^169^ which can act as recombination centers for excited electrons, and further accelerate the phonon‐assisted relaxation process. Taking BiInSe_3_ as an example,^110^, ^162^ it can absorb solar light over the entire solar spectrum (Figure 6b1). The electrons can be excited from conduction band to valence band and these photoexcited electrons can decay to the ground state in a spontaneous way (Figure 6b2). The abundant IB states in BiInSe_3_ enable strong electron–phonon interactions and the energy of electrons is transferred to the lattice. The process of phonon‐assisted decay of electrons is thus accelerated, resulting in efficient solar energy conversion.

#### 4.2.3 Dual‐Resonance Optical Mode

Among a large number of optical absorption materials, both the plasmonic and all‐dielectric nanomaterials have been widely investigated for solar photothermal conversion.^10^, ^112^, ^170^ For some metals or highly doped semiconductors, the optical absorption can be largely enhanced via LSPR, such as Au,^4^ Ag,^171^ Al,^112^ and Ti_2_O_3_.^6^ Although the absorption coefficients of high‐index all‐dielectric nanomaterials are much smaller than those of plasmonic ones,^172^, ^173^ they have been demonstrated to produce heat as those metals based on the phase retardation effect, such as Si and Ge nanoparticles.^111^, ^174^ Although the plasmonic and all‐dielectric nanomaterials possess their unique strength in solar energy conversion, there is no finding of nanophotonic materials combining the characters of both two kinds of nanomaterials for more efficient solar energy harvest.

A broadband absorber is fabricated using Te nanomaterials with wide light absorption covering the entire solar spectrum and high solar conversion performance.^109^ The unique permittivity mechanism of Te is established to interpret the high photothermal performance. Unlike Si and Au, the real part of permittivity of Te nanomaterials experiences a transition from negative (300 < λ < 490 nm) to positive (λ > 490 nm) (Figure 6c1). The dual optical property of Te nanomaterials is revealed by the scattering spectra. As shown in Figure 6c2, it presents a plasmonic‐like resonance when the size of Te nanomaterials is smaller than 120 nm. When the size of Te nanomaterials locates at the range of 120–340 nm, it appears to be a Mie‐type resonance and the resonance moves from visible to NIR region as the size increases (Figure 6c3). Thus, the total absorption of Te nanomaterials can be enhanced by both the resonances. These interesting findings suggest that the Te nanomaterials could be developed as a promising solar energy conversion material for photothermal evaporation.

#### 4.2.4 Hybrid Design

Through the assembly of 1D SWCNTs and 2D MoS_2_ nanosheets, the assembled film has an enhanced spectral absorption covering the whole solar spectrum ranging from 300 to 2500 nm, differing from the pure SWCNTs film.^151^ Moreover, the assembled film with a thickness of 6 nm can absorb 95% of the light and it is comparable to thick film with typical thicknesses range from several µm to tens of mm. Similar to the design of SWCNTs‐MoS_2_, the quasi‐1D material of cotton cloth can also be used to combine with 2D photothermal materials.^156^ The MoS_2_‐cotton cloth system exhibits a high light absorption covering the entire solar spectrum, which results from the large absorption surface of cotton cloth and light trapping effect in the hierarchically arranged MoS_2_ nanoflower shapes. Moreover, the path length of light can be increased owing to the light scattering of assembled materials, such as bacterial nanocellulose in hybridizing with MoS_2_,^157^ which further increases the absorption.

### 4.3 Photothermal Conversion

**Figure 7:**
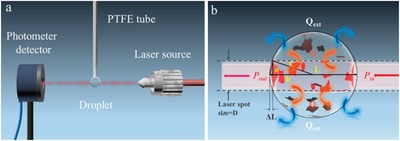
a) Droplet‐based experimental setup for determining photothermal conversion. b) Thermal equilibrium of droplet upon laser irradiation. Reproduced with permission.^37^ Copyright 2017, American Chemical Society.

After absorbing light energy, the rest of the work is how to convert the light energy into heat to the maximum. Unlike the absorption optimization with many investigations, the research on PTCE lack of efforts because some 2D photothermal materials possess a high PTCE in itself.^37^ Moreover, the PTCE needs to be measured through elaborately designing the equipment, which increases the research difficulty.^175^ Li et al. first reported the internal PTCE of Ti_3_C_2_ MXene by using a delicately designed light heating system using a water droplet (**Figure** **7**a) and a perfect energy conversion was found with PTCE to be up to 100% (Figure 7b).^37^ The high PTCE originated from its metallic properties owing to plasmonic effect.

The 2D photothermal nanomaterials used for photothermal cancer therapy are normally applied for photothermal evaporation, and thus the experiences in enhancing photothermal performance can learn from each other. It is worth to mention that although the photothermal effects of nanomaterials can be developed for both photothermal evaporation and tumor treatment, their specific requirements for photothermal materials are different. A strong absorption and high PTCE are both required for tumor therapy and evaporation applications. The difference is that a broadband absorption application and high PTCE matching wide solar spectrum of 295–2500 nm is required for evaporation, whereas the photothermal materials for tumor treatment require strong absorption and high PTCE in the two biological windows (750–1000 nm, 1000–1350 nm). Lasers of 808 and 1064 nm are commonly used for photothermal cancer therapy.^92^ The different needs in these two different photothermal applications determine their different design strategies.

## 5 Design Strategies for Photothermal Evaporation Performance

For efficient photothermal evaporation, single material design for high photothermal performance is insufficient. Further design of whole evaporation system is also crucial for enhancing photothermal evaporation performance. The design strategies toward high water flux, low heat loss, stable mechanical property, excellent environmental compatibility, and low cost will be presented.

### 5.1 High Water Flux

The membrane materials such as inorganic materials (NaA zeolite),^176^ poly(vinyl alcohol),^177^ and inorganic‐polymer hybrid materials (silica/PVA)^178^ can be used for pervaporation desalination. Although the high ion rejection has been obtained by these membranes, the water flux is normally lower than 10 L m^−2^ h^−1^. Therefore, novel work has been made to develop effective membranes with higher water flux.^179^ For a typical evaporation system, the solar absorber membrane is normally put on the top surface of the system, and the water source is pumped through the capillary effect from the bottom. However, a drawback for this structure is that the salt remains crystallized on the membrane surface in the process of evaporation, which would block the water channel and reduces the further water supply, resulting in the destruction of the membrane and significant decrease of the evaporation rate finally.^112^, ^180^ Unfortunately, the salt‐blocking problem has been rarely studied, and it has become a key scientific challenge for practical solar evaporation. Thus, an effective strategy needs to be proposed to solve this problem.

**Figure 8:**
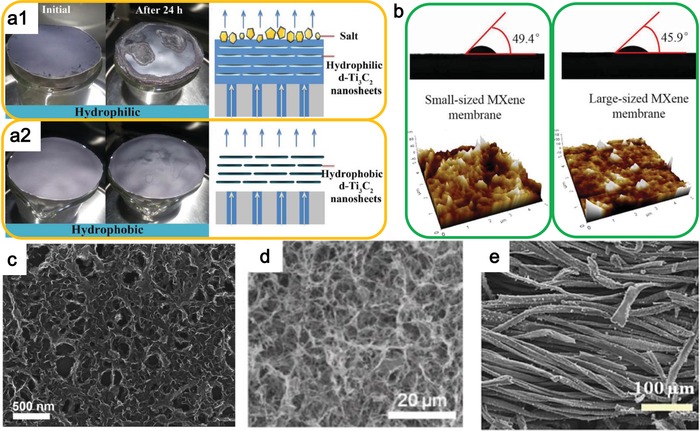
a) Comparison of the hydrophilic and hydrophobic membrane for photothermal evaporation. Reproduced with permission.^93^ Copyright 2018, Royal Society of Chemistry. b) Surface structures and water contact angles of small‐sized and large‐sized membrane. Reproduced with permission.^94^ Copyright 2017, Elsevier. c) SEM image of the SWCNT‐MoS_2_ porous film. Reproduced with permission.^151^ Copyright 2017, WILEY‐VCH. d) SEM image of the pristine BNC aerogel. Reproduced with permission.^157^ Copyright 2018, Elsevier. e) SEM image of the MoS_2_‐cotton cloth. Reproduced with permission.^156^ Copyright 2018, American Chemical Society.

Regarding this issue, Zhao et al. proposed a hydrophobic MXene membrane, composed of a salt‐blocking delaminated Ti_3_C_2_ nanosheet layer modified by trimethoxy silane for photothermal evaporation.^93^ For the hydrophilic Ti_3_C_2_ membrane (**Figure** **8**a1), the salt accumulated on the MXene membrane surface, leading to a gradually slower evaporation rate, while the surface of the hydrophobic Ti_3_C_2_ membrane remains almost clean after 24 h evaporation (Figure 8a2). For the membrane with hydrophilic nature, the membrane channels are blocked by the rested salt crystals and thus the vapor cannot be generated. However, the hydrophobic membrane prevents the water from infiltrating the Ti_3_C_2_ membrane and thus the blockage of salt is avoided. Then, the vapor can generate and go through the channels of stacked Ti_3_C_2_ nanosheets. Consequently, the hydrophobic surface and strong light absorption endow the Ti_3_C_2_ membrane with salt‐blocking ability and excellent photothermal conversion ability for effective and long‐term stable solar steam evaporation.

On the contrary, Liu et al. developed hydrophilic MXene membranes for pervaporation desalination.^94^ Considering the solution‐diffusion transport mechanism, the functional groups of —C=O and —OH on the MXene nanosheets would give the larger interlaminar spacing between the stacked MXene nanosheets, leading to effective molecular transport channels. Moreover, the hydrophilicity of MXene membranes made from large‐ and small‐sized MXene nanosheets is measured by water contact angle experiments. As illustrated in Figure 8b, the low water contact angle of the MXene stacks indicates a good hydrophilicity. Moreover, the water contact angle for the stacks composed of large‐sized MXene nanosheets (1–2 µm) is smaller than that composed of small‐sized MXene nanosheets (≈0.5 µm) owing to the different surface roughness, resulting in the higher water flux of small‐sized MXene membrane. Consequently, the novel MXene membrane exhibits high salt rejection (99.5%) and water flux (85.4 L m^−2^ h^−1^) at 65 °C due to the large interlayer spacing combining with hydrophilic surface. The high‐performing pervaporation desalination endows ultrathin 2D MXene membrane a bright future for photothermal evaporation applications.

In most of photothermal vaporization devices, the film is relatively thick to guarantee the high solar light absorption. However, the thick film would hinder the steam transport by these long pathways. Porous network structure is thus needed for fluent steam transport. SWCNTs are proved to have high breathability and permeability for efficient water vapor transport,^181^, ^182^, ^183^ and can be chosen to fabricate the hybrid system with 2D photothermal nanomaterials, such as MoS_2_.^151^ The thickness‐dependent water evaporation capability of SWCNTs‐MoS_2_ film is investigated. An evaporation saturation state can be observed as thickness increases because of the opposite effect of enhanced absorption and longer steam transport. The water evaporation efficiency of water can reach up to 91.5% at the power density of 5 kW m^−2^ by using SWCNT‐MoS_2_ film with ultrathin thickness of 6 nm which is comparable to most of localized heating systems with thicker film than 2 µm.^151^ This outstanding solar‐driven evaporation performance of the SWCNT‐MoS_2_ film benefits from both the excellent structure and thermal properties. Structurally, the ultrathin and sponge‐like SWCNTs structure leads to an excellent permeability that allows the unobstructed and rapid escape of the generated vapor from the localized heating site (Figure 8c). Additionally, the thermal‐conductive SWCNTs can transform the heat induced by MoS_2_ to water efficiently. Consequently, both the superior attributes of 2D MoS_2_ and 1D SWCNTs can be gathered to achieve an enhanced solar evaporation performance, which shows the promising combination strategy for nanomaterials with different dimensions.

Besides the synthetic inorganic materials, naturally occurring organic materials can also be used to transport water in an efficient way. Bacterial nanocellulose (BNC), as a polymer matrix secreted from bacteria, can entangle into a close‐packed network, which leads to high mechanical strength and porosity,^184^ and thus is chosen as the supporting materials for MoS_2_ (Figure 8d) _._ 157 With the high specific surface area and intrinsic hydrophilicity owing to the hydroxyl group loaded in BNC fibers, the water can be transported to the evaporation surface rapidly, resulting in high evaporation water flux. Similar to BNC, the 1D cotton cloth with large area can also be used to combine with MoS_2_ to make an efficient solar evaporator (Figure 8e).^156^ The hydrophilic surface of cotton cloth can guarantee a continuous water supply based on the capillary effect. The unique advantage of these natural organic fibers is the environmentally friendly property, which is much more suitable for treating environmental issues.

### 5.2 Thermal Management

It is well known that, besides material's intrinsic PTCE, photothermal evaporation performance can be significantly enhanced by two other factors, including maximizing light absorption through rationally designing surface structure and minimizing heat loss to bulk water through employing suitable heat barrier.^112^, ^185^, ^186^ Conventional photothermal systems suffer from severe energy loss to the bulk water because heating occurs at the interface of air and water.^187^ Therefore, floatable design is deployed for achieving efficient photothermal evaporation.

In the work Li et al., nonporous polystyrene foam is selected as heat barrier through attaching onto the back side of the solar absorber of MXene membrane as it contains no passageway and thus prevents the heat transfer to the ambient water environment while allowing water extraction up to the solar absorber from the circumjacent sides of the heat barrier. In the following, the elaborated design helps to concentrate the light‐induced heat at the interface between water and solar absorber of MXene membrane, resulting in an enhanced water evaporation efficiency.^63^ As expected, the photothermal evaporation efficiency of MXene‐polystyrene membrane was enhanced to be 84% under 1 sun irradiation (compared to 74% for MXene membrane), which makes it rank at the top performances in other reports.^5^, ^188^, ^189^, ^190^ CF is also chosen as a supporting material because of its high stability, light weight, and low thermal conductivity. Through combining with BiInSe_3_, a high evaporation performance can be obtained owing to the rational heat management.^162^ The porous nature of CF renders the weak thermal conductivity, which can thus efficiently impede heat transfer to the back side of the device and more heat energy locates at the water surface, avoiding the unnecessary heat loss to bulk water. In the meantime, the numerous channels in CF allow the water transport from the bulk water to the device surface owing to the evaporation‐enabled capillary effect.

### 5.3 Mechanical Stability

Besides high solar light absorption for the best use of solar energy, the good mechanical stability of supporting membranes also needs to be met because an ideal photothermal evaporation system should float on water surface and guarantee the membranes can be recycled in keeping a stable evaporation performance. Kinds of floating materials have been developed, such as the air‐laid paper,^191^ mixed cellulose ester,^62^ and the metallic floating materials, such as a 3D porous membrane.^112^ The paper‐based supporting systems are fragile while the metal‐based systems are strong and expensive.

**Figure 9:**
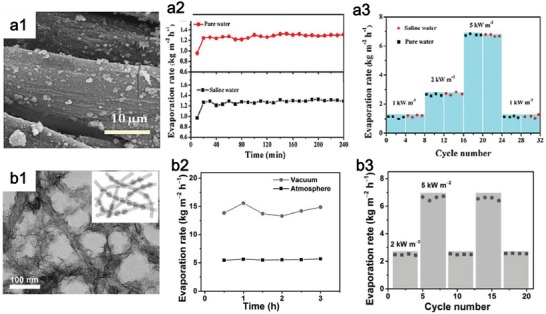
a) Stable evaporation performance of PMoS_2_‐cotton cloth film. Reproduced with permission.^156^ Copyright 2018, American Chemical Society. a1) High‐magnification SEM images of PMoS_2_‐cotton cloth. a2) Stable evaporation rate during 4 h. a3) Reusability during 32 cycles. b) Stable evaporation performance of SWCNT‐MoS_2_ hybrid film. Reproduced with permission.^151^ Copyright 2017, WILEY‐VCH. b1) SEM image of SWCNT‐MoS_2_. b2) Stable evaporation rate during 3 h. b3) Reusability during 20 cycles.

Cotton cloth can be used to attach MoS_2_ through the self‐growth technique in a hydrothermal process (**Figure** **9**a1), and the hydrophilic surface is further optimized through polyethylene glycol (PEG) modification.^156^ Stable evaporation kept after 4 h illumination (Figure 9a2) and after 32 cycles (Figure 9a3). Of course, the thick film could ensure a high mechanical stability, but the water flux can be reduced owing to long and winding steam transport, as discussed above. Up to date, the well‐designed synthesis of ultrathin photothermal film with high mechanical stability is still in the infancy. Owing to the unique tubular structure of the SWCNT and the close joint between the SWCNTs, SWCNTs‐based materials possess high mechanical strength.^152^, ^154^ Thus, the SWCNTs can be employed to combine with solar absorber materials to enhance the mechanical strength and stability of entire evaporation system. For example, through the inlay of the 2D photothermal nanomaterials of MoS_2_ on SWCNTs substrate (Figure 9b1), a SWCNT‐MoS_2_ film can be achieved with stable evaporation rate during 3 h and 20 cycles solar irradiation (Figure 9b2,b3).^151^ Consequently, the combination of high‐absorptive 2D nanomaterials with other mechanically stable materials provides an efficient strategy for improving stability in realizing the practical evaporation.

### 5.4 Stability of Photothermal Materials

**Figure 10:**
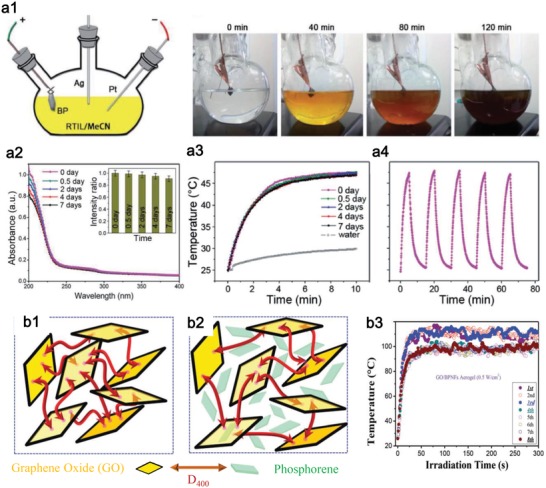
a) Fluorinated phosphorene for enhanced stability. Reproduced with permission.^192^ Copyright 2017, WILEY‐VCH. a1) Schematic of the atomistic fluorination experimental setup and time‐dependent exfoliation effect. a2) Stability of fluorinated phosphorene shown by the time‐dependent absorbance at ambient conditions for 7 d. a3) Time‐dependent photothermal heating curves. a4) Photothermal stability in five cycles. b) Graphene oxide/phosphorene with robust thermostability. Reproduced with permission.^193^ Copyright 2017, Royal Society of Chemistry. b1) Schematic of GO gelation with cross‐linked agent D400 for protecting b2) phosphorene. b3) Photothermal stability at high temperature during 8 d.

The material for photothermal evaporation is required to possess high stability during the service process on the one hand, and is demanded to degrade when leaked to water. The relatively stable Ti_3_C_2_ MXene^37^ and MoS_2_ 151 are very suitable for photothermal evaporation. Of course, if the stability of easy‐degradable materials can be enhanced through some doping or modification techniques, they can also be considered for photothermal evaporation. Tang et al. reported a facile strategy to obtain stable phosphorene through a fluorination process during electrochemical exfoliation (**Figure** **10**a1).^192^ The fluorinated phosphorene were observed to exhibit enhanced environmental stability owing to the antihydration and antioxidation effects of the introduction of highly electronegative fluorine adatoms. Of particular importance is the demonstration of the robust absorption (Figure 10a2) and photothermal stability over one week (Figure 10a3, a4). Xing et al. reported a physical phosphorene protection strategy by constructing a 3D hybrid aerogel using GO (Figure 10b1, b2), which results in an outstanding photothermal stability under a high photothermal‐induced temperature (>100 °C) (Figure 10b3).^193^ These techniques provide effective strategies to overcome the drawbacks of weak stability in phosphorene and endow phosphorene a great promise in photothermal evaporation. Of course, these strategies are feasible for other 2D degradable photothermal nanomaterials.

### 5.5 Environmental Influence

The basic principle for clean water production avoids the introducing of new environmental problems. Thus, the photothermal nanomaterials are required to be nontoxic and environmental‐friendly.^175^ Both MoS_2_ 122 and phosphorene^194^ were reported to have inappreciable toxicity. Mo is an essential element to the living body. Phosphorene can be degraded into nontoxic phosphate,^195^ which exists in living body and thus phosphorene is also well compatible.^196^

Through different surface modification, such as the soybean phospholipid, the Ti_3_C_2_,^2^ Nb_2_C,^92^ and Ta_4_C_3_ 91 MXenes exhibit excellent biocompatibility. However, one potential problem is from its hazardous fabrication process, which employs the etching of hydrofluoric acid (HF). The residual HF on MXene surface may bring serious toxicity to environment. Therefore, the development of fluorine‐free fabrication method for MXene is of particular importance for photothermal evaporation. Yu et al.^197^ and Xuan et al.^198^ proposed to use organic base intercalation method to exfoliate MXenes, and great biocompatibility was achieved.

Te nanomaterials were reported to be toxic,^107^ which may be caused by the toxic tellurates and tellurites from oxidation of elemental Te.^199^ To avoid the oxidation of Te and following toxicity, a nanoreactor made of hollow albumin is designed for protecting the growth of Te, and the prepared Te nanomaterials show high resistance ability to laser light.^108^ Then, there was no detectable damage to normal tissues, proving superior biocompatibility of Te nanomaterials for environmental applications.

The biotoxicity measurement for photothermal evaporation was only reported by Ghim et al. on MoS_2_ materials as far as we know.^157^ The cytotoxicity of MoS_2_ was proved to be lower than that of GO through using the model cell lines of mouse embryonic cells and human mammary cells. Thus, the employment of MoS_2_ in photothermal evaporation can mitigate the potential toxicity to the living entities in water in case of leakage or even the discard of the photothermal materials into water environment. This study should arouse a wide research interest on the potential risk of photothermal materials for practical photothermal evaporation.

Besides the photothermal materials, the environmental influences of supporting materials should also be considered. Naturally occurring materials, possessing inherent advantage of environmental‐friendly characteristics, have been investigated for supporting photothermal materials, such as the cotton cloth,^156^ bacterial nanocellulose,^157^ etc.

### 5.6 Cost

Normally, two different types of cost need to be considered for practical use. One is the cost of 2D photothermal materials and the other is the supporting material. Phosphorene, as one typical photothermal agent, has received extensive attention in the photothermal treatment of tumors due to its excellent biocompatibility, degradability, and high‐performance photothermal effect. However, there is no report on the application of phosphorene in the field of photothermal evaporation. This may be because of the degradability of phosphorene, which limits its long‐term existence in the water environment, as discussed above. On the other hand, it may be mainly because of its high cost, which does not meet the low‐cost principle of practical application of solar evaporation. The cost of supporting materials is also a key promotion factor toward the practical application of photothermal evaporation. Thus, some cheaper and abundant materials were chosen, such as the cotton cloth^156^ and polystyrene foam.^93^

## 6 Challenges and Perspectives

**Figure 11:**
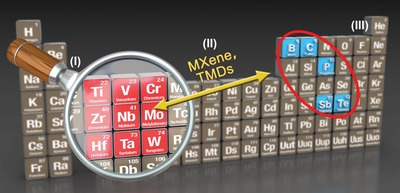
The current popular photothermal agents and potential photothermal agents in the future.

In general, three main kinds of 2D photothermal agents are reported up to now, layer Xenes, compound of nonmetal elements and early transition metal, and nonlayer early transition metals. Most of the elemental photothermal agents, i.e., layer Xenes, occur at the boundary of metal and nonmetal elements, including graphene, phosphorene, antimonene, tellurene, and borophene (**Figure** **11**). Most of them have not been investigated for photothermal evaporation. The compound of nonmetal elements and early transition metal includes two main kinds of 2D photothermal series, one is the MXene and the other is TMD. Titanium is the first reported nonlayer 2D photothermal agent among early transition metals with excellent photothermal performance and super biocompatibility (in press), motivating a large room in photothermal research on other early transition metals. These early transition metals possess the potential of plasmonic localized heating, which contributes to an efficient photothermal effect.

### 6.1 MXenes

The excellent photothermal performance of Ti_3_C_2_, Nb_2_C, and Ta_4_C_3_ endow the wide investigations in photothermal cancer therapy.^200^ Moreover, the abundant surface modification is promising for high evaporation water flux. Both attributes would lead to a bright future of MXene in photothermal evaporation. However, only the Ti_3_C_2_ MXene has been applied for photothermal evaporation up to date.^37^ The photothermal conversion efficiency of Ti_3_C_2_ MXene at 808 nm is measured to be 30.6%,^2^ while it is 100% upon irradiation at visible light, such as 473 or 785 nm,^37^ which just corresponds to the most intense solar spectrum, indicating the super ability of Ti_3_C_2_ in converting solar light energy. High photothermal efficiency of Nb_2_C and Ta_4_C_3_ upon irradiation at near‐infrared (NIR) light (808 or 1064 nm) has also been reported whereas the photothermal conversion ability in visible light has not been investigated. Moreover, the same fabrication technique of MXenes materials (etching and sonication) endows their rich surface properties and resulting high water flux proved by Ti_3_C_2_ MXenes, which is viable for other kinds of MXenes. Therefore, a large space for employing other kinds of MXenes in photothermal evaporation needs to be further explored owing to their high PTCE and beneficial surface groups.

### 6.2 Tellurene

Currently, the photothermal investigation of Te nanostructures is limited by the Te nanorods,^107^ Te nanodots,^108^ and Te nanoparticles,^109^ which limit the further exploration of its plentiful properties. In recent years, tellurene has been greatly developed in both fabrications and applications.^102^ The fabrication techniques mainly include van der Waals epitaxy (vdWE),^201^, ^202^, ^203^ liquid‐phase exfoliation,^41^ and solution‐based reduction method.^100^ Inspired by the fabrication techniques, the applications of tellurene involve field‐effect transistors,^100^ photoacoustic imaging guided photodynamic tumor therapy,^204^ photodetector,^41^, ^201^, ^205^ etc. However, there were few reports on tellurene‐based photothermal evaporation. The unique chain‐like structure of Te nanomaterial is distinct from other layer materials.^206^ The photothermal evaporation film is normally designed by combining 1D and 2D nanomaterials, such as SWCNT‐MoS_2_ hybrid film^151^ and MoS_2_‐cotton cloth film.^156^ This design can enhance the mechanical strength of whole evaporation film and increase the permeability and breathability for high evaporation water flux. The chain‐like structure of Te is prone to create 1D structure,^101^ which thus can be used as a template for fabricating other linear materials, including the MS_2_ (M = Re, Mo, W) nanotubes,^207^ Bi_2_Te_3_ nanotubes,^208^ TeCuPt alloy nanowires,^209^ etc. This fabrication technique can be employed to produce the Te‐based in situ growing 1D‐2D hybrid film for photothermal evaporation.

### 6.3 2D Group V Elements

A family of 2D Xenes, stemming from group‐VA layered materials (P, As, Sb, Bi), has aroused an increasing interest in theoretical work and practical applications.^210^, ^211^, ^212^, ^213^, ^214^ Different from 2D metallic group‐IIIA (borophene) materials,^215^, ^216^, ^217^, ^218^, ^219^, ^220^, ^221^, ^222^, ^223^, ^224^, ^225^ and 2D semimetallic group‐IVA (graphene, silicene, germanene, and stanene),^226^, ^227^, ^228^, ^229^ 2D nanostructured materials in group‐VA possess the properties of semiconductors with significant bandgaps, which is vital for some optoelectronics devices.

Some photothermal agents in 2D Group V elements, including phosphorene^20^ and antimonene,^18^, ^230^ have gained a great interest in photothermal cancer therapy owing to their efficient photothermal conversion, biocompatibility, and degradability, especially for phosphorene, but rare research for photothermal evaporation. The efficient photothermal conversion and biocompatibility will also be beneficial to the environmental application but the degradability is a drawback, which may result in the rare study work. However, numerous work has been attempted to fix this stability issue, leading to a bright future of 2D group V‐elements‐based photothermal evaporation.

#### 6.3.1 Phosphorene

Phosphorene is the first and also the most investigated one in 2D group‐VA family. In 2014, Li et al. first fabricated the phosphorene and built the phosphorene‐based field‐effect transistor.^231^ Then, the following studies reveal other promising properties, such as superior mechanical properties,^232^, ^233^ highly anisotropic carrier transport,^234^, ^235^, ^236^, ^237^, ^238^ negative Poisson's ratios,^239^ strain‐induced conduction bands,^240^ perpendicular topological character, and excellent optical responses.^235^, ^241^, ^242^ Moreover, the wrinkled structure of phosphorene enables it to possess larger area compared with the flat‐surfaced 2D materials and benefits its loading capability for drugs,^20^, ^243^ imaging agents,^244^, ^245^ metal ions,^246^ etc. All these above‐mentioned characteristics of BP make it suitable for numerous applications in transistors,^247^, ^248^, ^249^, ^250^, ^251^ batteries,^252^, ^253^, ^254^, ^255^ energy storage,^256^ photonics,^235^, ^257^, ^258^ sensors,^259^, ^260^, ^261^, ^262^ and photothermal cancer therapy.^20^, ^263^

Particularly, the bandgap of phosphorene is layer number dependent and can be tuned in the range of 0.3–2 eV,^78^ which plays an important role in filling up the bandgap blank of other 2D materials, such as low bandgap of MXene^264^ or the large bandgap of TMDs. The wide and adjustable bandgap endows phosphorene a wide optical response from ultraviolet (UV) to NIR regions, showing the wide absorption ability corresponding to solar spectrum. However, the successful application of phosphorene as photothermal agent in biomedical field have not led to its investigations in environmental applications, such as the photothermal evaporation, which may be because of its degradability.^265^ Fortunately, the stability of phosphorene can be enhanced through many techniques, including covalent bonding with carbon free radicals^266^ and aryl diazonium,^267^ intercalation of alkali metal hydrides,^268^ doping of fluorine adatoms,^269^ surface coordination of titanium sulfonate ligand,^266^ metal‐ion modification,^246^ and encapsulation by hexagonal BN,^270^, ^271^ graphene shell,^272^ graphene oxide,^193^ organic monolayers,^273^ AlO*_x_*,^247^, ^274^ etc. Of course, seeking other stable phosphorene‐analogue photothermal materials is also a possible method, such as the tin sulfide (SnS).^275^, ^276^, ^277^ These strategies would open a new potential for the photothermal application of phosphorene and phosphorene analogue materials in environmental issues.

#### 6.3.2 Antimonene

To obtain high stable and similar high performance of phosphorene, scientists have turned their eyes on the cousins of phosphorene in the same VA group, i.e., the arsenene,^278^, ^279^, ^280^, ^281^ antimonene,^211^, ^282^, ^283^ and bismuthene.^284^, ^285^ Zhang et al. predicted the wide bandgap of novel 2D semiconducting arsenene and antimonene.^278^ After that, numerous theoretical studies and fabrication techniques were developed.^210^, ^211^, ^212^, ^213^ However, only the photothermal effect of antimonene was reported.^18^ As expected, the antimonene achieves a higher stability than phosphorene in optical applications.^286^, ^287^ Antimonene has been widely investigated theoretically and is predicted to possess excellent thermal conductivity,^288^ good stability, superior carrier mobility,^289^ extraordinary spintronic properties,^290^ and strain‐induced band transition.^278^ These theoretical findings motivate the further experimental work on fabrication and application. On account of the ultrasmall layer gap and strong binding energy,^278^ it remains a challenge to fabricate intact and high‐quality antimonene. Recently, the experimental synthesis of antimonene has been demonstrated through liquid‐phase exfoliation,^39^ epitaxial growth,^291^ van der Waals epitaxy,^292^ and mechanical isolation.^293^ Beneficial from the successful fabrication, the applications were rapidly developed, includes the electrocatalysis,^294^ energy storage,^295^ supercapacitor,^296^ perovskite solar cells,^297^ and cancer therapy.^18^

In photothermal‐related applications, antimonene quantum dots (AMQDs) are reported to possess a high PTCE (45.5%), which is even higher than MXenes and phosphorene. However, the shortcomings of AMQDs are the weak photostability and low absorption. The degradability of AMQDs is assessed in the irradiation of near‐infrared light and the color of AMQDs solution becomes transparent after irradiation of tens of minutes. This performance is clearly not suitable for photothermal evaporation, which needs a super photostability. Through some calculation techniques, the stability of antimonene was predicted to be ameliorated through noncovalent functionalization of small molecules^298^ and introduction of Stone–Wales defects.^299^ Rare research is deployed to tackle the stability problem of antimonene, which may be due to its good stability in air and most of applications of antimonene is operated in air environment, such as the optoelectronics and thermoelectric applications.^282^, ^291^ In the future, some other stability enhancement tactics for unstable materials, such as the phosphorene, can be employed for antimonene. Moreover, the weak optical absorbance was also observed shown by the low extinction coefficient (5.58 L g^−1^ cm^−1^) in Tao's report.^18^ To enhance the absorption, some above‐mentioned strategies can be employed, for instance, the defect modulation^159^ and composite design.^151^ Moreover, AMQDs are used for biomedical application because of their efficient kidney clearance, while for photothermal evaporation, the larger sized antimonene in the form of nanosheet may be the better choice owing to their enhanced stability and stronger absorption compared with AMQDs. To take advantage of the high photothermal conversion ability of AMQDs, both weaknesses must be overcome.

### 6.4 Borophene

Elemental boron, located at the neighborhood of carbon, arouses a rising attention in its 2D form. Borophene, as the lightest 2D material up to now, possesses a series of structures with polymorphism and anisotropy,^300^, ^301^, ^302^ and contributes to numerous unique attributes, such as metallicity, optical transparency,^303^ mechanical flexibility,^304^, ^305^ etc. These integrated properties occurring in borophene are not found in other 2D members. Metallicity is the most distinct character compared with other semimetals of graphene and silicene or semiconductor of phosphorene. Thus, it is expected for optically transparent electrode promisingly.^303^ The conventional phonon‐mediated superconductivity is also predicted from its intrinsic metallicity and the strong electron–phonon coupling owing to the light atomic weight of boron.^306^, ^307^ Other than the metal property of borophene, it is interesting to find a strong photoluminescence emission band at ≈626 nm deriving from its direct bandgap (2.25 eV).^308^ Moreover, small bending stiffness coupled with low mass of borophene results in high phonon velocities, leading to efficient thermal transport.^309^

**Figure 12:**
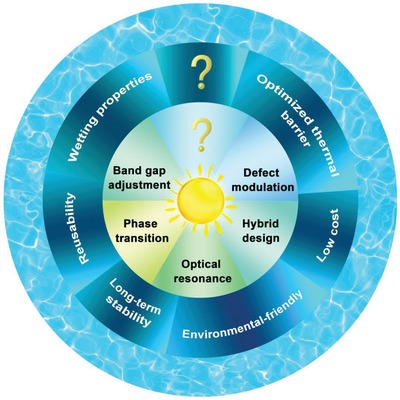
Strategies for practical photothermal evaporation.

Theoretically, it was expected that the borophene can grow on metal substrates, such as Ag(111), Cu(111),^310^ and Au(111),^301^ owing to the stabilization of the sp^2^ hybridization from metal passivation. The borophene was first demonstrated experimentally by Mannix et al. through growing on Ag (111) under ultrahigh‐vacuum conditions with anisotropic electronic properties and metallic characteristics.^311^ Feng et al. also used molecular beam epitaxy (MBE) technique to realize the growth of borophene.^312^ Different from these physical growth, Tai et al. synthesized atomically thin borophene on copper foils via chemical vapor deposition.^308^ More extraordinarily, Ji et al. employ the top‐down strategies to obtain borophene through combining thermal oxidation etching and liquid‐phase exfoliation.^313^ Following the development of fabrication technique of borophene, its application has been widely explored, including the anode material with high capacity for Li‐ion and Na‐ion batteries,^215^, ^216^ electrocatalyst for hydrogen evolution reaction,^314^ photothermal cancer therapy,^313^ etc. Unfortunately, the optical transparent borophene possesses the low absorption with extinction coefficient of ≈2.5 L g^−1^ cm^−1^,^313^ which greatly limits its further exploration for photothermal evaporation. The absorption of borophene appears to be a size‐dependent property and size of borophene can be optimized to obtain an enhanced absorption. Moreover, the metallic properties of borophene may endow its plasmonic heating through rationally designing the 2D structure. Owing to the nonlayer nature of bulk boron, the exfoliated borophene possess some dangling bonds, making it easily degradable, which needs to be further tackled. Finally, the exact strategies for enhancing the photothermal evaporation performance are proposed in mainly two avenues, one is optimizing the photothermal performance of 2D photothermal agents themselves and the other is enhancing the evaporation performance in view of evaporation system toward practical use (**Figure** **12**).

## 7 Conclusions

In conclusion, the recent progress of 2D nanomaterials‐based photothermal evaporation systems is presented. Based on the photothermal mechanism for various 2D nanomaterials, solar light absorption can be enhanced through the introduction of atomic vacancy, dual‐resonance optical mode, bandgap adjustment, phase transition, etc. Furthermore, the strategies for efficient photothermal evaporation system in terms of water flux, thermal supervision, and stability are discussed and some other properties, such as environmental influence and cost from the point of environment and commercial application are also evaluated. It is worth noting that new environmental and social problems should not be introduced in the process of solving the water crisis. Challenges of different 2D nanomaterials toward photothermal evaporation application are pointed out and corresponding strategies are proposed. In brief, the important role of 2D materials based‐photothermal evaporation in solving current water crisis is highlighted and more attention should be paid for the emerging green technology.

Excellent photothermal performance requires both a strong absorbance and a high PTCE and the research on enhancing PTCE needs to be strengthened in surveying the related reports. Of course, the weakness of most photothermal agents is the low absorption, like the GO (*k* = 3.6 L g^−1^ cm^−1^), antimonene *k* = 5.6 L g^−1^ cm^−1^), and borophene (*k* = 2.5 L g^−1^ cm^−1^), while some kinds of photothermal agents can obtain a fairly high extinction coefficient, for instance, phosphorene (14.8 L g^−1^ cm^−1^), WS_2_ (23.8 L g^−1^ cm^−1^), MoS_2_ (28.4 L g^−1^ cm^−1^), Ti_3_C_2_ (25.2 L g^−1^ cm^−1^), and Nb_2_C (36.4 L g^−1^ cm^−1^), which are several times of the antimonene and borophene. However, the PTCE of most 2D photothermal agents locates at a similar level (28–45%), for instance, 28.4% for phosphorene, 30.6% for Ti_3_C_2_ MXene, 42.5% for borophene, and 45.5% for antimonene. Thus, there is a large room for improving the absorption ability. That is why most of research focuses on increasing absorbance while less on photothermal conversion. Currently, different strategies for optimizing absorbance were employed for various kinds of photothermal agents, including defect modulation, bandgap adjustment, etc. These strategies can be combined to implement on one promising photothermal agent.

It is also interesting to find that new photothermal agent with a lower absorption ability always holds a higher photothermal conversion ability, indicating the independent absorption process and conversion process. Thus, according to the different photothermal performance evaluated by the two parameters of extinction coefficient and PTCE, it is impossible for choosing better photothermal agent because of the opposite absorption ability and photothermal conversion ability. To combine the absorption ability and conversion ability of one photothermal agent, a new parameter is proposed, namely, ePTCE, which is defined as the product of extinction coefficient and PTCE. Through the definition of this novel parameter, the photothermal performance can be comprehensively evaluated, for example, the phosphorene in Xenes with ePTCE of 4.2 L g^−1^ cm^−1^, Nb_2_C in MXenes family with ePTCE of 13.7 L g^−1^ cm^−1^, and MoSe_2_ in TMDs with ePTCE of 8.1 L g^−1^ cm^−1^. Overall speaking, the binary‐enes, including the MXenes and TMDs, possess a higher photothermal performance than Xenes and MXene holds the highest photothermal performance.

For improving the photothermal performance, most of the investigations focus on how to enhance the absorption but rare work on the PTCE enhancement. For the absorption, the current research employs the limited strategies, such as the defect engineering, bandgap adjustment, hybrid design, etc. Fortunately, many methods have been carried out to enhance the absorption in other solar light applications, which can be further followed. For example, surface plasmon resonance (SPR) has been widely investigated to lock photon and enhance absorption of graphene, yet for other 2D photothermal materials. Thus, SPR‐enhanced photothermal performance can be further explored and the plasmonic materials are proposed to integrate with some 2D semiconducting materials.

Up to now, the 2D materials‐based evaporation system is just in the early stage and focuses on the materials design. Further investigations can move toward structure design. For example, Li et al. elaborately design a cylindrical vapor generator structure, which can guarantee the lower temperature than that of surrounding environment, thus facilitating the energy absorption from the environment. The photothermal evaporation efficiency is observed to be up to 100%.^315^ Xu et al. employ mushrooms to achieve efficient evaporation because of the unique natural structure of umbrella‐shaped black pileus and porous context.^316^ This study points out the direction of next‐generation high‐efficient 2D materials based evaporation system, which needs more efforts on structure design.

Besides the most concerned photothermal performance, the stability and toxicity issues are also crucial especially in late‐stage research of practical applications, but lacking comprehensive studies. Generally, binary compounds, such as MXenes and MoS_2_, are more stable than 2D Xenes, including tellurene, Group VA‐enes of phosphorene and antimonene. Thus, the stability problem of Xenes needs to be highlighted for their further consideration. Up to now, more investigations about the protection strategies are developed on phosphorene, which can be further employed for enhancing the stability of other unstable photothermal Xenes. Both physical and chemical methods can be employed to enhance the stability of the unstable 2D materials, including encapsulation by other stable materials, noncovalent functionalization, doping, covalent functionalization, etc. Encapsulation by other hydrophilic/hydrophobic polymers is an efficient strategy because it can not only enhance the stability of photothermal agent but also optimize the surface properties to improve the evaporation water flux. In the meantime, the stability strategy should also consider the environmental influence and the cost. The fabrication should not introduce toxic element and be easily operable, and the additional materials to protect the unstable 2D materials should also be nontoxic and low cost. Some natural materials can be better choice for this need, such as the cotton cloth.

A basic and important principle is that new environmental problems should not be introduced in solving the environmental problem of water shortage. So far, very less report in photothermal evaporation field focuses on this topic. Therefore, besides the above‐mentioned photothermal performance and stability issues, the environmental consideration of photothermal agents also needs to be highlighted. Some biocompatible data of photothermal agents are available from the reports on photothermal cancer therapy. The MXenes and MoS_2_ with stable properties exhibit no toxicity to mouse, while some elemental materials with unstable properties, such as the tellurene, present the toxicity. The biocompatibility test can illustrate the environmental influence to some extent. However, the targeted research of photothermal agents on environmental influences should be further conducted toward the practical use. The toxicity of some photothermal agents is reported to be reduced through encapsulating by some nontoxic polymers but limited in biological environments and short period. Long‐term environmental tests, such as in saline seawater, may result in the falloff of encapsulated polymers and exposure of toxic photothermal agents, which needs to be further confirmed.

Each kind of photothermal material has its own advantage and disadvantage. Materials hybrid can be an efficient strategy to overcome the disadvantage of each other. For typical carbon‐based materials, there are many advantages in cost and photothermal performance. But some of them were reported to be toxic, such as the graphene. However, some natural products can be good carbon sources with low environmental influence, such as the mushrooms,^316^ artificial trees,^317^ etc. The natural carbon materials have low cost, low environmental influence, rational thermal management, and unhindered water supply passage. The 2D materials possess the strong absorption and high photothermal conversion efficiency. The combination of these two kinds of materials can make full use of the advantage of each other and promote the practical photothermal evaporation.

Solar energy utilization is the most promising way for solving energy challenge. In the process of photothermal evaporation, solar energy harvest is an efficient way to solve two main global challenges of energy and water in the meantime, thus enhancing the utilization efficiency of photothermal evaporation system. Gao et al. use the plasmonic gold nanoflowers to realize the solar vaporization. In the meanwhile, the triboelectricity can be harvested from the process of water condensate.^44^, ^318^ Ding et al. employ thermogalvanic and pyroelectric effect to harvest the solar‐induced heat energy through a hybrid photothermal generator.^319^ Li et al. store and recycle steam enthalpy from photothermal‐induced water steam.^15^ These investigations point out the further opportunities of photothermal evaporation for dealing with water and energy challenge in an efficient way.

## Conflict of Interest

The authors declare no conflict of interest.
